# Efficacy of Preoperative Oral Clonidine in Spine Surgery: A Systematic Review and Meta-Analysis of Randomized Controlled Trials

**DOI:** 10.3390/jcm15135270

**Published:** 2026-07-06

**Authors:** Ahmed Abu-Zaid, Abdulrahman Emad AlAyyaf, Maznah M. Alajmi, Abdulmuhsen Alqallaf, Batoul H. Aljaber, Waleed Bader Alazemi, Abdullah Khaled Alothainah, Mohammad F. Al-Mutairi, Mohammad Ali Behbehani, Khaled Mohammad Altamimi, Saud Jaber Almarri, Abdullah M. Alharran

**Affiliations:** 1 Department of Biochemistry and Molecular Medicine, College of Medicine, Alfaisal University, Riyadh 11533, Saudi Arabia; 2 Department of Clinical Sciences, College of Medicine, University of Sharjah, Sharjah 27272, United Arab Emirates; 3 Kuwait Institute for Medical Specializations, Kuwait City 12050, Kuwait; 4 Faculty of Medicine, University of Jordan, Amman 11942, Jordan; 5 Faculty of Medical Sciences, Newcastle University, Newcastle Upon Tyne NE1 7RU, UK; 6 College of Medicine and Medical Sciences, Arabian Gulf University, Manama 26671, Bahrain; 7 Mubarak Al-Kabir Hospital, Jabriya 47060, Kuwait

**Keywords:** bleeding, clonidine, meta-analysis, pain, spine surgery

## Abstract

**Introduction**: This study presents the first systematic review and meta-analysis of randomized controlled trials (RCTs) evaluating the efficacy and safety of oral clonidine in spinal surgery to guide clinical practice and inform future research. **Methods**: RCTs were identified through four databases (PubMed, Scopus, Web of Science, Cochrane Central) up to August 2025. Risk of bias was assessed, and intraoperative and postoperative outcomes were extracted. Data were pooled using mean differences (MD) or risk ratios (RR) with 95% confidence intervals. **Results**: Eight RCTs with 474 patients (237 clonidine, 237 control) were included. Four studies had a low risk of bias, while two had some concerns, and two had a high risk. Clonidine had no impact on surgery duration but significantly reduced blood loss (MD = −177.76 mL), mean arterial pressure (MD = −11.33 mmHg), and heart rate (MD = −21.18 bpm). Regarding postoperative outcomes, clonidine was associated with improved analgesia, including lower pain scores up to 12 h and a longer time to first analgesic request, with no significant difference in pain scores at 24 h or total postoperative opioid consumption. No significant increase in postoperative complications, including bradycardia, dizziness, headache, or visual disturbances, was observed. Surgeon satisfaction was higher in the clonidine group. Despite high heterogeneity in some outcomes, sensitivity analyses confirmed the robustness of most findings. **Conclusions**: Prophylactic oral clonidine in spinal surgery significantly reduces blood loss, heart rate, and mean arterial pressure, with added analgesic benefits and good tolerability. Further high-quality RCTs are needed to confirm these results in broader patient populations.

## 1. Introduction

Spinal surgery is frequently associated with significant intraoperative bleeding, presenting considerable challenges to both surgeons and anesthesiologists. Key contributors to this blood loss include wide incisions, lengthy surgical procedures, and the extensive handling of cancellous bone. Excessive bleeding during spinal surgery can complicate the surgical field, increase the risk of transfusion-related complications, prolong operative time, and adversely affect patient outcomes [1,2,3].

Effective control of bleeding remains a paramount surgical goal to ensure optimal visualization, reduce perioperative morbidity, and improve overall patient safety [4]. Various antifibrinolytic pharmacologic agents have been investigated for their hemostatic properties in spinal surgery, including aminocaproic acid [5], tranexamic acid [6], and aprotinin [7]. Despite their efficacy, these agents carry potential side effects and limitations that necessitate exploring alternative or adjunctive therapies.

In addition to blood loss, postoperative pain is another major concern in spinal surgery, often resulting in prolonged recovery times, increased opioid use, and delayed mobilization [8]. Effective pain management is therefore critical, not only for patient comfort but also for enhancing recovery and reducing postoperative complications [9].

Clonidine, a centrally acting alpha-2 adrenergic agonist, has emerged as a potential anti-hemorrhagic agent due to its ability to reduce sympathetic outflow, leading to decreased arterial blood pressure and heart rate. These effects contribute to a reduction in surgical bleeding by lowering blood flow to the operative site [10,11]. Clonidine also has well-documented analgesic properties mediated through spinal and supraspinal mechanisms, making it a useful adjunct for postoperative pain control [12]. Furthermore, clonidine’s pharmacokinetic profile, characterized by a relatively long half-life (~7.5 to 7.7 h) and favorable central nervous system (blood–brain barrier) penetration [13], makes it an attractive candidate for perioperative management in spinal surgeries.

Several randomized controlled trials (RCTs) have evaluated the use of clonidine during spinal surgery, demonstrating varying degrees of efficacy in reducing blood loss and improving hemodynamic stability. However, these studies are limited by small sample sizes, heterogeneity in clonidine dosing regimens, differences in patient populations, variability in surgical techniques, and inconsistent outcome measures [14,15,16,17,18,19,20,21,22]. To date, no comprehensive meta-analysis has synthesized the existing evidence on the efficacy and safety of clonidine in spinal surgery. Given the clinical importance of effective blood loss management and the potential benefits of clonidine, a systematic review and meta-analysis are warranted to provide a clear and evidence-based assessment.

The aim of this study is to conduct the first systematic review and meta-analysis of RCTs assessing the efficacy and safety of clonidine during spinal surgery, thereby informing clinical practice and guiding future research.

## 2. Methods

### 2.1. Study Protocol and Registration

This systematic review and meta-analysis were conducted in accordance with the Preferred Reporting Items for Systematic Reviews and Meta-Analyses (PRISMA) guidelines [23] and the recommendations outlined in the Cochrane Handbook for Systematic Reviews of Interventions [24]. Ethical approval was not necessary, as the review is based solely on previously published clinical trials. The protocol was registered in the PROSPERO database (identifier: CRD420251143509). Table S1 depicts the PRISMA checklist.

### 2.2. Eligibility Criteria

RCTs were eligible for inclusion if they adhered to the following Population, Intervention, Control, and Outcome (PICO) framework: adults (>18 years) undergoing spinal surgery were considered the target population; the intervention was oral clonidine administered at any dose; the control group received either a placebo or no intervention; and the outcomes of interest included intraoperative measures such as duration of surgery, estimated blood loss, heart rate, and mean arterial pressure, as well as postoperative measures including pain, total analgesic consumption, time to first analgesic request, complication rate, and surgeon satisfaction score. Studies were excluded if they compared oral clonidine with active interventions such as dexmedetomidine, investigated non-oral routes of administration (e.g., epidural, intravenous), were quasi-randomized, or were published solely as conference abstracts, proceedings, observational studies, in vitro studies, or reviews.

### 2.3. Data Sources and Search Strategy

A comprehensive systematic literature search was performed on 29 August 2025 across multiple electronic databases, including PubMed, Scopus, Web of Science, and the Cochrane Central Register of Controlled Trials. The search strategy combined keywords related to both the surgical procedure and the intervention: (“spine surgery” OR “spinal surgery” OR “spine fusion” OR “spine fusion surgery” OR “lumbar spine surgery” OR “lumbar spine fusion surgery” OR “lumbar fusion surgery” OR “discectomy” OR “laminectomy” OR “lumbar laminectomy” OR “spondylosyndesis” OR “lumbar fusion” OR “thoracolumbar” OR “lumbosacral” OR “spinal decompression” OR “diskectomy”) AND (clonidine OR catapres OR catapresan OR catapressan OR clonidin OR clonidina OR “alpha-2 adrenergic agonist*” OR “α2 agonist*”). A detailed summary of the search terms and results for each database is presented in Table S2. Additionally, an informal supplementary search was performed using Google Scholar to identify potentially relevant grey literature and additional studies. Moreover, the reference lists of relevant trials were manually screened to ensure the comprehensiveness of the search and to minimize the risk of omitting eligible studies.

### 2.4. Study Selection Methods

The screening and selection process was performed by two independent reviewers. All retrieved articles underwent a two-stage screening procedure. In the first phase, titles and abstracts were assessed for relevance. In the second phase, the full texts of potentially eligible studies were reviewed in detail. Any discrepancies between the reviewers were resolved through discussion until a consensus was reached. The same approach was applied during the risk of bias and statistical assessment to ensure the accuracy and reliability of the included studies.

### 2.5. Risk of Bias, Publication Bias, and Certainty of Evidence

The risk of bias in the included RCTs was assessed using the Cochrane Risk of Bias Tool 2 (RoB-2) [25]. This tool evaluates potential sources of bias across five domains: the randomization process, deviations from intended interventions, missing outcome data, measurement of outcomes, and selection of the reported results. Based on these assessments, each study was categorized as having a low risk of bias, some concerns, or a high risk of bias.

Assessment of publication bias was not feasible in this review. Egger’s test for funnel plot asymmetry has been shown to be unreliable when fewer than 10 studies are included in a meta-analysis [26]; therefore, we did not apply this method.

Certainty of evidence was assessed according to the Grading of Recommendations Assessment, Development, and Evaluation (GRADE) approach [27].

### 2.6. Data Items and Review Outcomes

A pilot data extraction was performed following the retrieval of full texts from eligible studies in order to design a structured Excel extraction form. The form consisted of three main sections: (i) summary characteristics of the included trials, (ii) baseline characteristics of the participants, and (iii) outcome data. The summary section captured essential study information, including the first author’s name, year of publication, country, study design, sample size, intervention details, surgical procedure, and type of anesthesia. The baseline section recorded group-specific characteristics, such as the number of participants, age, sex, body mass index (BMI), and American Society of Anesthesiologists (ASA) physical status classification.

The clinical outcomes assessed were divided into intraoperative and postoperative endpoints. Intraoperative measures included duration of surgery (minutes), estimated blood loss (mL) assessed using gravimetric and/or volumetric methods, mean heart rate (bpm), and mean arterial pressure (mmHg). Postoperative outcomes included pain scores measured on a 10-point visual analogue scale (VAS), total analgesic consumption during the first 24 h (irrespective of analgesic type), time to first analgesic request (minutes), complication rates (e.g., bradycardia, dizziness, headache, and visual disturbance), and surgeons’ satisfaction score, which was rated on a 3-point scale (good, fair, poor).

### 2.7. Effect Measures and Meta-Analysis

Dichotomous outcomes were analyzed using the Mantel–Haenszel method, and results were expressed as risk ratios (RRs) with 95% confidence intervals (CIs). For continuous outcomes, the results were summarized as mean differences (MDs) with 95% CIs. A fixed-effects model was used as the primary analytical approach; however, a random-effects model was applied in the presence of substantial heterogeneity, defined as a Chi-square test *p*-value < 0.1 combined with an I^2^ statistic > 50%. Sensitivity analyses were performed to test the robustness of the results by sequentially excluding one RCT at a time and recalculating the pooled effect size. Statistical significance was determined at a *p*-value < 0.05. All analyses were conducted using STATA software (version 18, StataCorp, College Station, TX, USA). For studies reporting outcomes at multiple time points, the last and most clinically relevant time point was selected. When outcome data were presented only in graphical form, values were extracted manually by two independent reviewers. A subgroup analysis was performed for postoperative pain scores (2–24 h).

## 3. Results

### 3.1. Systematic Review Search and Eligibility Process

**Figure 1:**
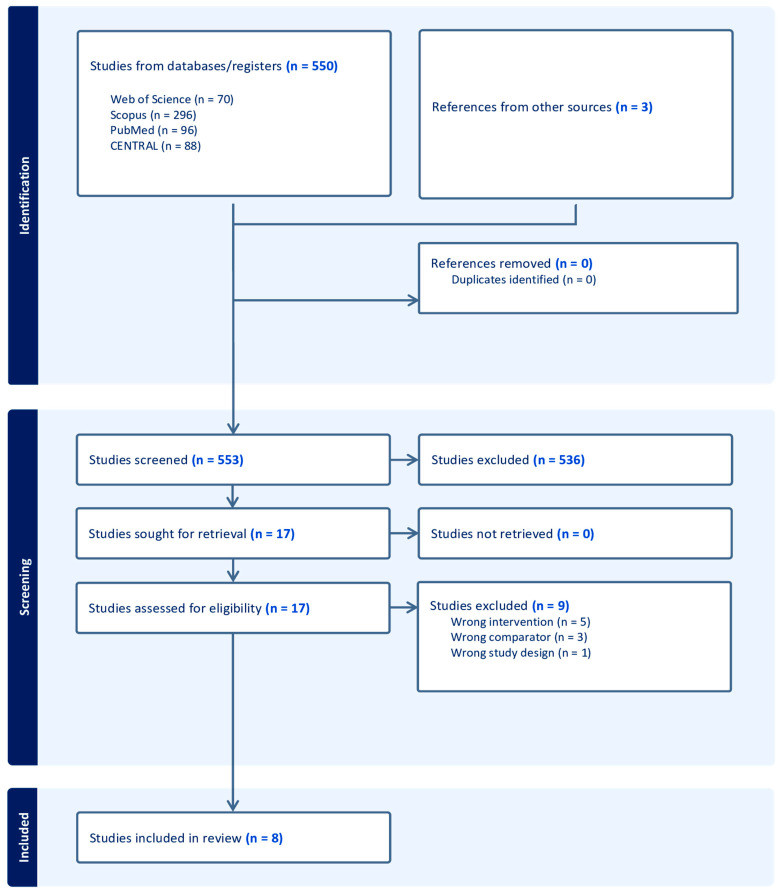
PRISMA flow diagram illustrates the systematic study selection process. PRISMA = preferred reporting items for systematic reviews and meta-analyses. The PRISMA checklist presented in the Supplementary Materials.

The systematic search initially identified 550 records across the following databases: Web of Science (*n* = 70), Scopus (*n* = 296), PubMed (*n* = 96), and Cochrane Central Register of Controlled Trials (*n* = 88). In addition, three studies were identified through manual reference screening. Following the initial screening, 17 full-text articles were assessed for eligibility. Of these, nine were excluded for not meeting the predefined PICO criteria, as depicted in Table S3. Ultimately, eight RCTs [14,15,16,17,18,19,20,21] satisfied the eligibility criteria and were included in the review, as depicted in Figure 1.

### 3.2. Summary and Baseline Characteristics of the Included Trials

A total of 474 participants were included across eight RCTs [14,15,16,17,18,19,20,21], with 237 assigned to the clonidine group and 237 to the control group. The studies were conducted in Iran, Egypt, and India. Most of the included trials involved posterior lumbar spine fusion performed under inhalational anesthesia (IA), and one RCT used total intravenous anesthesia (TIVA). In the majority of RCTs (*n* = 7), oral clonidine was administered at a dose of 100–200 μg, given 60–120 min prior to surgery; however, one study (Aezi et al., 2023) [18] reported the use of a 4 mg oral dose as per the original trial protocol. The detailed summary characteristics of the included RCTs are presented in Table 1. The majority of trials enrolled middle-aged participants. All studies that reported postoperative pain outcomes employed the 10-point VAS for pain assessment, while estimated blood loss was measured using gravimetric and volumetric methods. Baseline characteristics of the included RCTs and participants are provided in Table 2, and the anesthetic protocols used in each trial are summarized in Table S4.

### 3.3. Summary of Risk of Bias Assessment and Certainty of Evidence

**Figure 2:**
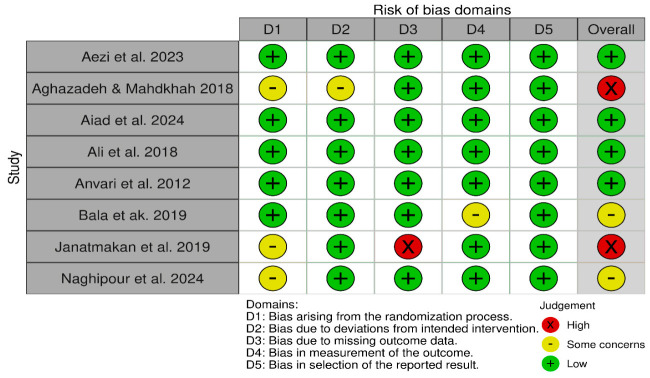

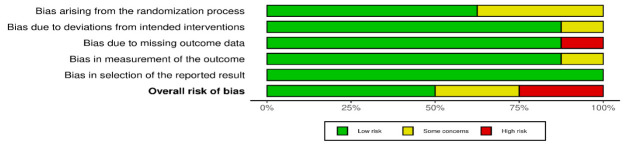
Risk of bias graph and summary of the included randomized controlled trials [14,15,16,17,18,19,20,21].

In terms of methodological quality, four RCTs were judged to have a low risk of bias, two were assessed as having some concerns, and two were considered to have a high risk of bias, Figure 2. Aghazadeh & Mahdkhah were rated as having some concerns in domains D1 and D2 due to insufficient details on the randomization process and blinding, suggesting a potential risk of selection bias despite balanced baseline characteristics. Bala et al. [17] were assessed as having some concerns in domain D4 because the blinding status of outcome assessors was not reported. Similarly, Janatmakan et al. [16] and Naghipour et al. [19] were rated as having some concerns in domain D1 owing to limited information regarding randomization and blinding, raising the possibility of selection bias. Moreover, Janatmakan et al. [16] were additionally judged to have a high risk of bias in domain D3, as a significant number of patients were excluded from the final analysis due to complications, suggesting attrition bias. The certainty of evidence for all outcomes ranged from very low to low because of concerns regarding risk of bias, inconsistency across studies, imprecision due to small sample sizes and wide confidence intervals, and the limited number of included trials for several outcomes.

### 3.4. Duration of Surgery (Minutes)

**Figure 3:**
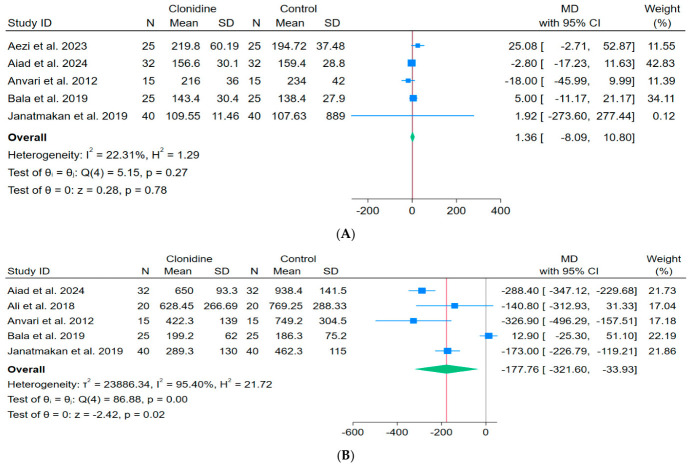
Meta-analysis of the (**A**) duration of surgery (minutes), and (**B**) estimated blood loss (mL). MD = mean difference; CI = confidence interval [14,15,16,17,18,20].

The pooled results demonstrated no significant difference in the duration of surgery between the clonidine and control groups (*n* = 274 patients, *n* = 5 RCTs, MD = 1.36 min, 95% CI [−8.09, 10.80], *p* = 0.78; Figure 3A). Statistical heterogeneity was low (I^2^ = 22.3%). Leave-one-out sensitivity analyses further confirmed the robustness of the pooled findings across all scenarios, Figure S1A.

### 3.5. Estimated Blood Loss (mL)

Preoperative oral clonidine was associated with a significant reduction in EBL compared with the control group (*n* = 264 patients, *n* = 5 RCTs, MD = −177.76 mL, 95% CI [−321.60, −33.93], *p* = 0.02; Figure 3B). However, statistical heterogeneity was considerable (I^2^ = 95.4%). Leave-one-out sensitivity analyses indicated that the overall pooled estimate became non-significant when either Anvari et al. [14] or Janatmakan et al. [16] was excluded, Figure S1B.

### 3.6. Heart Rate (Bpm)

**Figure 4:**
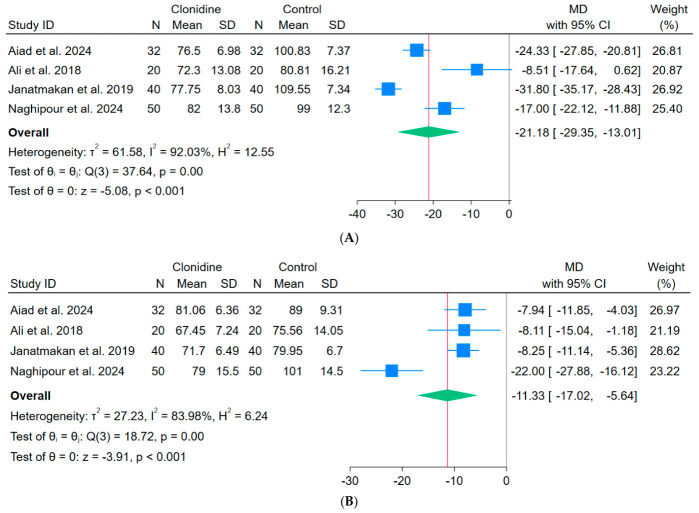
Meta-analysis of the intraoperative (**A**) heart rate (bpm), and (**B**) mean arterial pressure (mmHg). MD = mean difference; CI = confidence interval [15,16,19,20].

Preoperative oral clonidine was associated with a significant reduction in heart rate compared with the control group (*n* = 284 patients, *n* = 4 RCTs, MD = −21.18 bpm, 95% CI [−29.35, −13.01], *p* < 0.001; Figure 4A). However, statistical heterogeneity was considerable (I^2^ = 92.03%). Leave-one-out sensitivity analyses further confirmed the robustness of the pooled findings across all scenarios, Figure S2A.

### 3.7. Mean Arterial Pressure (mmHg)

Preoperative oral clonidine was associated with a significant reduction in MAP compared with the control group (*n* = 284 patients, *n* = 4 RCTs, MD = −11.33 mmHg, 95% CI [−17.02, −5.64], *p* < 0.001; Figure 4A). However, statistical heterogeneity was considerable (I^2^ = 83.98%). Leave-one-out sensitivity analyses further confirmed the robustness of the pooled findings across all scenarios, Figure S2B.

### 3.8. Postoperative Pain Score (10-Point VAS)

**Figure 5:**
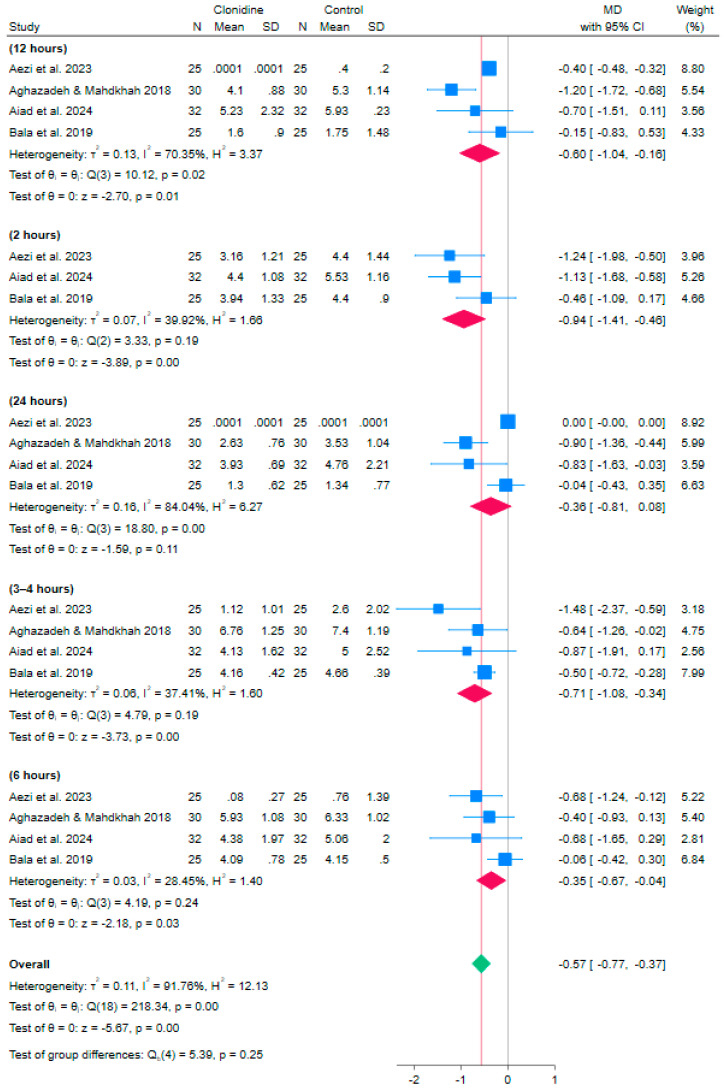
Meta-analysis of the postoperative pain score (10-point VAS). MD = mean difference; CI = confidence interval [17,18,20,21].

Preoperative oral clonidine was associated with a significant reduction in the overall pain score compared with the control group (*n* = 224 patients, *n* = 4 RCTs, MD = −0.57, 95% CI [−0.77, −0.37], *p* < 0.001; Figure 5). However, statistical heterogeneity was considerable (I^2^ = 91.76%). According to the subgroup analysis, preoperative oral clonidine was associated with a significant reduction compared with the control group in postoperative pain after 2 h (*n* = 3 RCTs, MD = −0.94, 95% CI [−1.41, −0.46], *p* < 0.001; Figure 5), after 3–4 h (*n* = 4 RCTs, MD = −0.71, 95% CI [−1.08, −0.34], *p* < 0.001; Figure 5), after 6 h (*n* = 4 RCTs, MD = −0.35, 95% CI [−0.67, −0.04], *p* = 0.03; Figure 5), and after 12 h (*n* = 4 RCTs, MD = −0.60, 95% CI [−1.04, −0.16], *p* = 0.01; Figure 5). However, the pooled results demonstrated no significant difference in the postoperative pain score after 24 h between the clonidine and control groups (*n* = 4 RCTs, MD = −0.36, 95% CI [−0.81, 0.08], *p* = 0.11; Figure 5).

### 3.9. Total Postoperative Opioid Consumption

**Figure 6:**
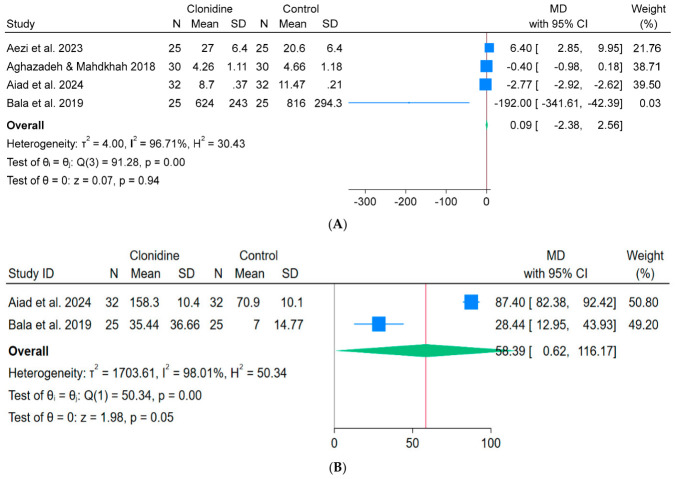
Meta-analysis of the (**A**) total postoperative opioid consumption (MME), and (**B**) time to 1st analgesia (minutes). All postoperative opioid consumption values were converted to MME prior to pooling to ensure consistency across studies reporting different opioid types. CI = confidence interval; MD = mean difference; MME = morphine milligram equivalent [17,18,20,21].

Preoperative oral clonidine was not associated with a statistically significant difference in total postoperative opioid consumption compared with the control group (*n* = 224 patients, *n* = 4 RCTs, MD = 0.09, 95% CI [−2.38, 2.56], *p* = 0.94; Figure 6A). However, statistical heterogeneity was considerable (I^2^ = 96.71%). Leave-one-out sensitivity analyses further confirmed the robustness of the pooled findings across all scenarios, Figure S2C.

### 3.10. Time to First Analgesia (Minutes)

Preoperative oral clonidine was associated with a significantly longer time to the first analgesic request compared with the control group (*n* = 114 patients, *n* = 2 RCTs, MD = 58.39 min, 95% CI [0.62, 116.17], *p* = 0.05; Figure 6B). However, statistical heterogeneity was considerable (I^2^ = 98.01%). Leave-one-out sensitivity analyses were not reliable for this outcome since they included only two RCTs.

### 3.11. Postoperative Complication Rate (%)

**Figure 7:**
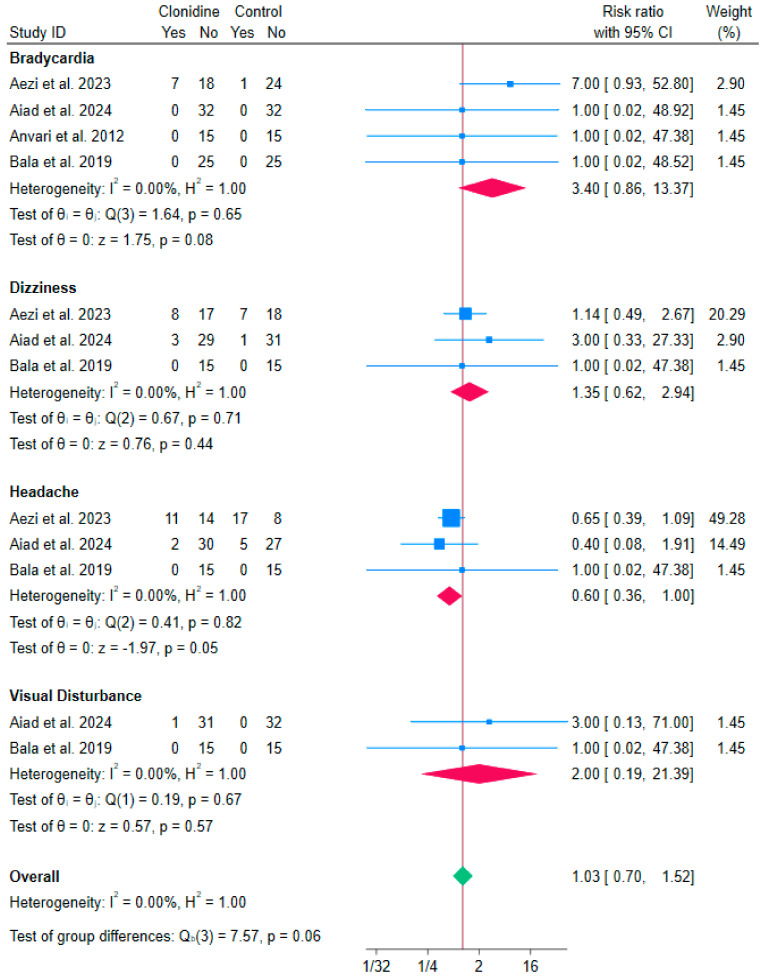
Meta-analysis of the rate of postoperative complications. CI = confidence interval [14,17,18,20].

The pooled results demonstrated no significant difference between the clonidine and control groups in terms of postoperative bradycardia rate (*n* = 194 patients, *n* = 4 RCTs, RR = 3.40, 95% CI [0.86, 13.37], *p* = 0.08, Figure 7), dizziness rate (*n* = 144 patients, *n* = 3 RCTs, RR = 1.35, 95% CI [0.62, 2.94], *p* = 0.44, Figure 7), headache rate (*n* = 144 patients, *n* = 3 RCTs, RR = 0.60, 95% CI [0.36, 1.00], *p* = 0.05, Figure 7), and visual disturbance rate (*n* = 94 patients, *n* = *n* = 2 RCTs, RR = 2.00, 95% CI [0.19, 21.39], *p* = 0.57, Figure 7). Statistical heterogeneity was nil (I^2^ = 0%).

### 3.12. Surgeons’ Satisfaction Score (3-Point Scale)

Preoperative oral clonidine was associated with significantly higher surgeon satisfaction in the good category compared with the control group (*n* = 134 patients, *n* = 3 RCTs, RR = 1.59, 95% CI [1.25, 2.04], *p* = 0.0002; Figure S3). In contrast, no significant difference was observed between groups in the fair category (*n* = 134 patients, *n* = 3 RCTs, RR = 0.53, 95% CI [0.27, 1.03], *p* = 0.06; Figure S3). For the poor category, preoperative oral clonidine was associated with significantly lower ratings compared with the control group (*n* = 134 patients, *n* = 3 RCTs, RR = 0.39, 95% CI [0.18, 0.86], *p* = 0.02; Figure S3). Statistical heterogeneity was low (I^2^ < 50%).

## 4. Discussion

### 4.1. Summary of Principal Findings

We conducted this systematic review and meta-analysis to assess the clinical efficacy and safety of prophylactic oral clonidine in patients undergoing spinal surgery. Eight RCTs, comprising 474 patients (clonidine group = 237; control group = 237), met the eligibility criteria and were included in the final analysis. The included trials demonstrated variable methodological quality: four RCTs were rated as having low risk of bias, while the remainder had some concerns (*n* = 2) or high risk (*n* = 2). The meta-analysis showed that preoperative oral clonidine significantly reduced intraoperative blood loss, mean arterial pressure, and heart rate compared with control interventions. Furthermore, clonidine was associated with significantly lower postoperative pain scores at multiple time points and a longer time to first analgesic request. No significant differences were found between groups in total postoperative opioid consumption, surgery duration, or length of hospital stay. Regarding safety, clonidine was generally well tolerated. Although no statistically significant differences were observed in postoperative complications overall, the bradycardia outcome (RR = 3.40, 95% CI 0.86–13.37) showed a notably elevated point estimate with a wide confidence interval, suggesting substantial imprecision and possible underpowering due to low event rates and small sample sizes. Therefore, a clinically relevant increase in risk cannot be excluded, and these safety findings should be interpreted with caution. Surgeon satisfaction ratings favored the clonidine group, with significantly higher ratings in the “good” category and fewer ratings in the “poor” category. Sensitivity analyses confirmed the robustness of the pooled estimates for most outcomes, though the effect on estimated blood loss became non-significant when specific studies were excluded.

However, these findings should be interpreted with caution given that the certainty of evidence ranged from very low to low according to the GRADE framework, mainly due to risk of bias, inconsistency, and imprecision. Therefore, the observed benefits should be considered preliminary and hypothesis-generating rather than definitive.

### 4.2. Interpretation of Results and Clinical Implications

Spinal surgery is an inherently aggressive and invasive procedure, often associated with considerable intraoperative blood loss due to large incisions, prolonged operative time, and the vascularity of spinal and paraspinal tissues. Achieving effective control of blood loss remains a key surgical priority to ensure a clear operative field, reduce transfusion needs, and improve patient outcomes [1,2,3]. In this context, patients undergoing spinal surgery may reasonably benefit from the prophylactic use of hemostatic agents.

Clonidine, an α2-adrenergic agonist, represents a promising candidate owing to its dual mechanism of action. Its sympatholytic properties attenuate the release of catecholamines, thereby lowering heart rate and mean arterial pressure [10,11]—parameters closely linked to controlled hypotension or hypotensive anesthesia [28]. This hemodynamic modulation has been shown to significantly reduce intraoperative blood loss, as demonstrated in the present meta-analysis. Beyond its hemostatic effect, clonidine also exerts clinically meaningful analgesic benefits [12]. These were reflected in our pooled results, which revealed significantly lower postoperative pain scores at multiple time points (2, 3–4, 6, and 12 h), reduced total analgesic consumption, and a prolonged time to first analgesic request compared to control. Together, these findings suggest that preoperative clonidine not only enhances intraoperative hemodynamic stability but also contributes to improved postoperative pain control and a potential reduction in opioid requirements—key factors in promoting early recovery and enhancing overall surgical outcomes.

Clonidine has previously demonstrated efficacy in minimizing blood loss across various surgical disciplines, including acetabular fracture surgery [29], endoscopic sinus surgery [30], open rhinoplasty [31], and cesarean section [32]. The findings of this meta-analysis further expand the clinical utility of clonidine by supporting its role in spinal surgery. The observed reduction in intraoperative blood loss and postoperative hemoglobin drop—without a corresponding increase in major adverse events—highlights clonidine’s potential as a safe, cost-effective, and multipurpose adjunct in perioperative care for spine surgery patients.

These findings are particularly relevant in enhanced recovery after surgery (ERAS) protocols, where minimizing surgical stress, reducing blood loss, and optimizing analgesia are key principles [33]. The sedative [34] and analgesic [12] effects of clonidine may further contribute to smoother postoperative recovery, reduced opioid consumption, and shorter hospital stays. Given its relatively low cost, favorable safety profile, and multiple perioperative benefits, clonidine represents a promising pharmacologic option that warrants consideration in standardized spinal surgery protocols.

### 4.3. Strengths and Limitations

Our study possesses several notable strengths that merit emphasis. To our knowledge, this is the inaugural systematic review and meta-analysis evaluating the efficacy and safety of prophylactic clonidine compared with passive control (placebo or no treatment) in the context of spinal surgery. By restricting our analysis to RCTs, we aimed to ensure robust, evidence-based conclusions derived from high-quality study designs. The included RCTs were generally of low risk of bias (>50%), enhancing the overall credibility and reliability of the findings. We also reported a wide range of clinically relevant efficacy and safety endpoints, providing a more complete understanding of clonidine’s perioperative role. In addition, the robustness of our conclusions was reinforced through sensitivity analyses, including leave-one-out meta-analysis.

Nonetheless, our study has several limitations that should be acknowledged. The limited number of included RCTs and their relatively small sample sizes constitute major constraints, potentially affecting the generalizability of the findings. Additionally, the fact that most of the studies were conducted in Iran may further limit their broader applicability. Some outcome measures demonstrated notable heterogeneity, which may be attributed to variations in perioperative protocols—including differences in clonidine dosing, type of anesthesia, surgical techniques, and patient characteristics. These clinical differences may have influenced the pooled effect estimates. The substantial heterogeneity observed in outcomes such as estimated blood loss and heart rate further supports this variability and highlights the inconsistency in hemodynamic and surgical responses across trials. Importantly, sensitivity analyses revealed that the statistically significant reduction in estimated blood loss was not robust, as the pooled effect became non-significant when individual studies (particularly Anvari et al. [14] and Janatmakan et al. [16]) were excluded, indicating that this outcome may be driven by specific studies rather than a consistent effect across all trials. Formal subgroup analyses based on clonidine dose and anesthetic technique were not performed in the present meta-analysis due to the very small number of included studies and the highly unbalanced distribution of key variables (dose: 5 studies at 200 μg, 1 study at 150 μg, 1 study at 100 μg, and 1 study at 4 mg; anesthesia: 6 inhalational anesthesia, 1 total intravenous anesthesia, and 1 not reported). Under these conditions, subgroup analyses would be statistically underpowered and potentially misleading, and therefore were not considered methodologically appropriate.

Although we intended to evaluate publication bias using funnel plots, this was not feasible due to the small number of studies in each individual meta-analysis, as at least 10 studies are required for a meaningful assessment [26].

### 4.4. Future Directions

In light of the limitations identified in the current evidence base, future investigations should prioritize conducting additional multicenter, large-scale, and well-designed RCTs comparing prophylactic clonidine to placebo in patients undergoing spinal surgery. Several factors are known to influence intraoperative blood loss during spinal procedures, including the surgical approach, extent of instrumentation, duration of surgery, and the anatomical level of the spine involved. Due to the limited number of included RCTs, subgroup analyses based on these critical variables could not be performed in the present review. Prospective studies are warranted to evaluate the differential efficacy of clonidine based on specific surgical techniques (e.g., minimally invasive vs. open approaches) or types of spinal pathology. Additionally, further research could explore the comparative effectiveness of different routes of clonidine administration—such as oral, intravenous, or neuraxial—and their respective impacts on intraoperative blood loss, hemodynamic stability, and postoperative pain outcomes. Moreover, future studies might investigate the potential additive benefits of clonidine when used in combination with other pharmacologic agents, such as dexmedetomidine or local anesthetics, to enhance perioperative outcomes. Also, identifying patient subgroups that are most likely to benefit from clonidine—based on factors such as baseline blood pressure, pain sensitivity, or surgical complexity—may help to individualize its use and optimize its clinical utility in spinal surgery. Lastly, future trials should also adopt standardized perioperative protocols, including uniform clonidine dosing regimens, anesthetic techniques, and clearly defined surgical and analgesic pathways, in order to reduce methodological heterogeneity and improve comparability across studies.

## 5. Conclusions

Prophylactic oral clonidine in spinal surgery significantly reduces heart rate and mean arterial pressure, while also providing meaningful analgesic benefits. Although a reduction in intraoperative blood loss was observed, this finding should be interpreted with caution due to the sensitivity of individual studies in the pooled analysis. In addition, oral clonidine was well tolerated, with no increase in serious adverse events or cardiovascular complications. Further high-quality RCTs are needed to confirm these findings across broader patient populations.

## Figures and Tables

**Table 1:** Summary information of the included randomized controlled trials.

Study ID	Study Design	Country	Trial Duration	Sample Size, *n*	Trial Arms		Surgical Procedure	Type of Anesthesia
					Intervention	Control		
Aezi et al. 2023 [18]	RCT	Iran	Not reported	50	Oral clonidine 4 mg, 60 min before the surgery *	Placebo	Spine surgery (posterolateral fusion of lumbar spine)	IA
Aghazadeh & Mahdkhah 2018 [21]	RCT	Iran	Not reported	60	Oral clonidine 200 μg, 60 min before the surgery	Placebo	Spine surgery (lumbar disc herniation surgery)	Not reported
Aiad et al. 2024 [20]	RCT	Egypt	August 2018-June 2020	64	Oral clonidine 200 μg, 90 min before the surgery	Placebo	Spine surgery (posterior fusion of lumbar spine)	IA
Ali et al. 2018 [15]	RCT	Egypt	April 2016–April 2017	40	Oral clonidine 100 μg, 120 min before the surgery	Placebo	Spine surgery (spine fusion surgery)	IA
Anvari et al. 2012 [14]	RCT	Iran	Not reported	30	Oral clonidine 200 μg, 90 min before the surgery	Placebo	Spine surgery (posterior fusion of lumbar spine)	TIVA
Bala et al. 2019 [17]	RCT	India	Not reported	50	Oral clonidine 150 μg, 90 min before the surgery	Placebo	Spine surgery (thoracolumbar spinal surgery)	IA
Janatmakan et al. 2019 [16]	RCT	Iran	Not reported	80	Oral clonidine 200 μg, 90 min before the surgery	Placebo	Spine surgery	IA
Naghipour et al. 2024 [19]	RCT	Iran	January 2020–July 2020	100	Oral clonidine 200 μg, 60 min before the surgery	No treatment	Spine surgery (laminectomy with pedicle screw fixation)	IA

RCT: randomized controlled trial; IA: inhalational anesthesia; TIVA: total intravenous anesthesia. * Aezi et al. 2023 [18] administered oral clonidine at a dose of 4 mg, whereas the remaining studies used doses ranging from 100 to 200 μg.

**Table 2:** Baseline characteristics of the included participants and studies.

Study ID	Group	*n*	Age (Years)	Sex, *n* [Male/Female]	ASA, *n* [I/II]	BMI (kg/m^2^)	Pain Score	Blood Loss Measurement
Aezi et al. 2023 [18]	Clonidine	25	51.64 ± 12.70	Not reported	Not reported	29.87 ± 3.16	10-point Visual Analog Scale	Not reported
	Placebo	25	49.44 ± 11.82	Not reported	Not reported	29.64 ± 4		
Aghazadeh & Mahdkhah 2018 [21]	Clonidine	30	20–65 (range)	Not reported	Not reported	Not reported	10-point Visual Analog Scale	Not reported
	Placebo	30	20–65 (range)	Not reported	Not reported	Not reported		
Aiad et al. 2024 [20]	Clonidine	32	53.1 ± 6.2	[12/20]	[17/15]	30 ± 2.9	10-point Visual Analog Scale	Gravimetric and volumetric
	Placebo	32	52.4 ± 6.7	[15/17]	[16/16]	29.1 ± 3.8		
Ali et al. 2018 [15]	Clonidine	20	18–60 (range)	Not reported	Not reported	Not reported	Not reported	Gravimetric and volumetric
	Placebo	20	18–60 (range)	Not reported	Not reported	Not reported		
Anvari et al. 2012 [14]	Clonidine	15	44 ± 13.9	[9/6]	[13/2]	Not reported	Not reported	Gravimetric and volumetric
	Placebo	15	36.4 ± 11.5	[11/4]	[10/5]	Not reported		
Bala et al. 2019 [17]	Clonidine	25	34.08 ± 14.34	[17/8]	[21/4]	21.97 ± 2.76	10-point Visual Analog Scale	Not reported
	Placebo	25	39.76 ± 12.05	[13/12]	[20/5]	23.51 ± 4.20		
Janatmakan et al. 2019 [16]	Clonidine	40	39.9 ± 8.37	[11/29]	[33/7]	Not reported	Not reported	Gravimetric and volumetric
	Placebo	40	42 ± 4.54	[8/32]	[34/6]	Not reported		
Naghipour et al. 2024 [19]	Clonidine	50	41.6 ± 3.39	[60/40]	[88/12]	27.5 ± 1.92	Not reported	Not reported
	Control	50	43.6 ± 6.73			27.3 ± 2.05		

ASA: American Society of Anesthesiology; BMI: body mass index.

## Data Availability

All data are available within the manuscript and its Supplementary Materials.

## Supplementary Materials

The following supporting information can be downloaded at: https://www.mdpi.com/article/10.3390/jcm15135270/s1, Table S1: The PRISMA 2020 checklist; Table S2: Detailed search strategy for each database during the systematic search phase; Table S3: List of excluded studies during the full-text screening step; Table S4: Detailed summary of anesthesia and surgical protocols of the included trials; Figure S1: Leave-one-out sensitivity analysis of the (A) duration of surgery (minutes), and (B) estimated blood loss (mL); Figure S2: Leave-one-out sensitivity analysis of the intraoperative (A) heart rate (bpm), (B) mean arterial pressure (mmHg), and (C) total analgesic consumption; Figure S3: Meta-analysis of the surgeons’ satisfaction score (3-points). CI = confidence interval.
