# Supplementary material for: Efficacy of Preoperative Oral Clonidine in Spine Surgery: A Systematic Review and Meta-Analysis of Randomized Controlled Trials
Source: J Clin Med. 2026 Jul 6;15(13):5270. doi: 10.3390/jcm15135270 (PMC13363613; doi:10.3390/jcm15135270)

SUPPLEMENTARY FILE

SUPPLEMENTARY TABLE LEGENDS

Table S1. The PRISMA 2020 checklist.

Table S2. Detailed search strategy for each database during the systematic search phase.

Table S3. List of excluded studies during the full-text screening step.

Table S4. Detailed summary of anesthesia and surgical protocols of the included trials.

SUPPLEMENTARY FIGURES LEGENDS

Figure S1. Leave-one-out sensitivity analysis of the **[A]** duration of surgery (minutes), and **[B]** estimated blood loss (mL).

Figure S2. Leave-one-out sensitivity analysis of the intraoperative **[A]** heart rate (bpm), **[B]** mean arterial pressure (mmHg), and **[C]** total analgesic consumption.

Figure S3. Meta-analysis of the surgeons' satisfaction score (3-points). CI = confidence interval.

Table S1. The PRISMA 2020 checklist.

Section and Topic	Item #	Checklist item	Location where item is reported
**TITLE**			
Title	1	Identify the report as a systematic review.	1
**ABSTRACT**			
Abstract	2	See the PRISMA 2020 for Abstracts checklist.	1
**INTRODUCTION**			
Rationale	3	Describe the rationale for the review in the context of existing knowledge.	2
Objectives	4	Provide an explicit statement of the objective(s) or question(s) the review addresses.	2
**METHODS**			
Eligibility criteria	5	Specify the inclusion and exclusion criteria for the review and how studies were grouped for the syntheses.	3
Information sources	6	Specify all databases, registers, websites, organisations, reference lists and other sources searched or consulted to identify studies. Specify the date when each source was last searched or consulted.	3
Search strategy	7	Present the full search strategies for all databases, registers and websites, including any filters and limits used.	3
Selection process	8	Specify the methods used to decide whether a study met the inclusion criteria of the review, including how many reviewers screened each record and each report retrieved, whether they worked independently, and if applicable, details of automation tools used in the process.	3
Data collection process	9	Specify the methods used to collect data from reports, including how many reviewers collected data from each report, whether they worked independently, any processes for obtaining or confirming data from study investigators, and if applicable, details of automation tools used in the process.	4
Data items	10a	List and define all outcomes for which data were sought. Specify whether all results that were compatible with each outcome domain in each study were sought (e.g. for all measures, time points, analyses), and if not, the methods used to decide which results to collect.	4
	10b	List and define all other variables for which data were sought (e.g. participant and intervention characteristics, funding sources). Describe any assumptions made about any missing or unclear information.	4
Study risk of bias assessment	11	Specify the methods used to assess risk of bias in the included studies, including details of the tool(s) used, how many reviewers assessed each study and whether they worked independently, and if applicable, details of automation tools used in the process.	4
Effect measures	12	Specify for each outcome the effect measure(s) (e.g. risk ratio, mean difference) used in the synthesis or presentation of results.	4
Synthesis methods	13a	Describe the processes used to decide which studies were eligible for each synthesis (e.g. tabulating the study intervention characteristics and comparing against the planned groups for each synthesis (item #5)).	4
	13b	Describe any methods required to prepare the data for presentation or synthesis, such as handling of missing summary statistics, or data conversions.	4
	13c	Describe any methods used to tabulate or visually display results of individual studies and syntheses.	4
	13d	Describe any methods used to synthesize results and provide a rationale for the choice(s). If meta-analysis was performed, describe the model(s), method(s) to identify the presence and extent of statistical heterogeneity, and software package(s) used.	4
	13e	Describe any methods used to explore possible causes of heterogeneity among study results (e.g. subgroup analysis, meta-regression).	4
	13f	Describe any sensitivity analyses conducted to assess robustness of the synthesized results.	4
Reporting bias assessment	14	Describe any methods used to assess risk of bias due to missing results in a synthesis (arising from reporting biases).	3
Certainty	15	Describe any methods used to assess certainty (or confidence) in the body of evidence for an outcome.	4

Section and Topic	Item #	Checklist item	Location where item is reported
assessment			
**RESULTS**			
Study selection	16a	Describe the results of the search and selection process, from the number of records identified in the search to the number of studies included in the review, ideally using a flow diagram.	4
	16b	Cite studies that might appear to meet the inclusion criteria, but which were excluded, and explain why they were excluded.	4
Study characteristics	17	Cite each included study and present its characteristics.	5
Risk of bias in studies	18	Present assessments of risk of bias for each included study.	5
Results of individual studies	19	For all outcomes, present, for each study: (a) summary statistics for each group (where appropriate) and (b) an effect estimate and its precision (e.g. confidence/credible interval), ideally using structured tables or plots.	5-7
Results of syntheses	20a	For each synthesis, briefly summarise the characteristics and risk of bias among contributing studies.	5-7
	20b	Present results of all statistical syntheses conducted. If meta-analysis was done, present for each the summary estimate and its precision (e.g. confidence/credible interval) and measures of statistical heterogeneity. If comparing groups, describe the direction of the effect.	5-7
	20c	Present results of all investigations of possible causes of heterogeneity among study results.	5-7
	20d	Present results of all sensitivity analyses conducted to assess the robustness of the synthesized results.	5-7
Reporting biases	21	Present assessments of risk of bias due to missing results (arising from reporting biases) for each synthesis assessed.	5
Certainty of evidence	22	Present assessments of certainty (or confidence) in the body of evidence for each outcome assessed.	5
**DISCUSSION**			
Discussion	23a	Provide a general interpretation of the results in the context of other evidence.	7-8
	23b	Discuss any limitations of the evidence included in the review.	8
	23c	Discuss any limitations of the review processes used.	8
	23d	Discuss implications of the results for practice, policy, and future research.	8-9
**OTHER INFORMATION**			
Registration and protocol	24a	Provide registration information for the review, including register name and registration number, or state that the review was not registered.	2
	24b	Indicate where the review protocol can be accessed, or state that a protocol was not prepared.	2
	24c	Describe and explain any amendments to information provided at registration or in the protocol.	2
Support	25	Describe sources of financial or non-financial support for the review, and the role of the funders or sponsors in the review.	19
Competing interests	26	Declare any competing interests of review authors.	19
Availability of data, code and other materials	27	Report which of the following are publicly available and where they can be found: template data collection forms; data extracted from included studies; data used for all analyses; analytic code; any other materials used in the review.	19

Table S2. Detailed search strategy for each database during the systematic search phase.

Database	Search Strategy	Filter	Results
**PubMed**	(“spine surgery” OR “spinal surgery” OR “spine fusion” OR “spine fusion surgery” OR “lumbar spine surgery” OR “lumbar spine fusion surgery” OR “lumbar fusion surgery” OR “discectomy” OR “laminectomy” OR “lumbar laminectomy” OR “Spondylosyndesis” OR “lumbar fusion” OR “thoracolumbar” OR “lumbosacral” OR “spinal decompression” OR “diskectomy”) AND (clonidine OR catapres OR catapresan OR catapressan OR clonidin OR clonidina OR clonidã OR "clonidine hydrochloride" OR clonidinhydrochlorid OR clonidini OR clonidinum OR clonigen OR clonistada OR clonnirit OR clophelinum OR dixarit OR duraclon OR edolglau OR haemiton OR hypodine OR hypolax OR iporel OR isoglaucon OR jenloga OR kapvay OR klofelino OR kochaniin OR lonid OR melzin OR menograine OR normopresan OR paracefan OR pinsanidine OR "run rui" OR winpress OR arkamin OR aruclonin OR atensina OR catapin OR chianda OR chlofazoline OR chlophazolin OR "clonid-ophtal" OR "alpha-2 adrenergic agonist*" OR "α2 agonist*")	All Fields	n = 96
**CENTRAL**	(“spine surgery” OR “spinal surgery” OR “spine fusion” OR “spine fusion surgery” OR “lumbar spine surgery” OR “lumbar spine fusion surgery” OR “lumbar fusion surgery” OR “discectomy” OR “laminectomy” OR “lumbar laminectomy” OR “Spondylosyndesis” OR “lumbar fusion” OR “thoracolumbar” OR “lumbosacral” OR “spinal decompression” OR “diskectomy”) AND (clonidine OR catapres OR catapresan OR catapressan OR clonidin OR clonidina OR clonidã OR "clonidine hydrochloride" OR clonidinhydrochlorid OR clonidini OR clonidinum OR clonigen OR clonistada OR clonnirit OR clophelinum OR dixarit OR duraclon OR edolglau OR haemiton OR hypodine OR hypolax OR iporel OR isoglaucon OR jenloga OR kapvay OR klofelino OR kochaniin OR lonid OR melzin OR menograine OR normopresan OR paracefan OR pinsanidine OR "run rui" OR winpress OR arkamin OR aruclonin OR atensina OR catapin OR chianda OR chlofazoline OR chlophazolin OR "clonid-ophtal" OR "alpha-2 adrenergic agonist*" OR "α2 agonist*")	Title Abstract Keyword-Trials	n = 88
**Web of Science**	(“spine surgery” OR “spinal surgery” OR “spine fusion” OR “spine fusion surgery” OR “lumbar spine surgery” OR “lumbar spine fusion surgery” OR “lumbar fusion surgery” OR “discectomy” OR “laminectomy” OR “lumbar laminectomy” OR “Spondylosyndesis” OR “lumbar fusion” OR “thoracolumbar” OR “lumbosacral” OR “spinal decompression” OR “diskectomy”) AND (clonidine OR catapres OR catapresan OR catapressan OR clonidin OR clonidina OR clonidã OR "clonidine hydrochloride" OR clonidinhydrochlorid OR clonidini OR clonidinum OR clonigen OR clonistada OR clonnirit OR clophelinum OR dixarit OR duraclon OR edolglau OR haemiton OR hypodine OR hypolax OR catapin OR chianda OR chlofazoline OR chlophazolin OR "alpha-2 adrenergic agonist*" OR "α2 agonist*")	All Fields	n = 70
**Scopus**	(“spine surgery” OR “spinal surgery” OR “spine fusion” OR “spine fusion surgery” OR “lumbar spine surgery” OR “lumbar spine fusion surgery” OR “lumbar fusion surgery” OR “discectomy” OR “laminectomy” OR “lumbar laminectomy” OR “Spondylosyndesis” OR “lumbar fusion” OR “thoracolumbar” OR “lumbosacral” OR “spinal decompression” OR “diskectomy”) AND (clonidine OR catapres OR catapresan OR catapressan OR clonidin OR clonidina OR clonidã OR "clonidine hydrochloride" OR clonidinhydrochlorid OR clonidini OR clonidinum OR clonigen OR clonistada OR clonnirit OR clophelinum OR dixarit OR duraclon OR edolglau OR haemiton OR hypodine OR hypolax OR iporel OR isoglaucon OR jenloga OR kapvay OR klofelino OR kochaniin OR lonid OR melzin OR menograine OR normopresan OR paracefan OR pinsanidine OR "run rui" OR winpress OR arkamin OR aruclonin OR atensina OR catapin OR chianda OR chlofazoline OR chlophazolin OR "clonid-ophtal" OR "alpha-2 adrenergic agonist*" OR "α2 agonist*")	Article title, Abstract, Keywords	n = 296

Table S3. List of excluded studies during the full-text screening step.

Study ID	Title	Reason of exclusion
Birkebæk et al. 2025	Intraoperative Clonidine in Spine Surgery: A Randomised Controlled Trial	Wrong intervention – IV clonidine
Birkebæk et al. 2024	Intraoperative clonidine in endometriosis and spine surgery: A protocol for two randomised, blinded, placebo-controlled trials	Wrong study design – protocol
Dhas et al. 2023	Assessment of haemodynamic response to tracheal intubation and prone positioning following clonidine and enalaprilat in lumbar spine surgeries: A double blind randomised controlled trial	Wrong comparison – enalaprilat
Nagappa et al. 2018	Clonidine as an Adjuvant to Caudal Epidural Ropivacaine for Lumbosacral Spine Surgeries	Wrong intervention – epidural clonidine
Mariappanet al. 2014	Comparing the effects of oral clonidine premedication with intraoperative dexmedetomidine infusion on anesthetic requirement and recovery from anesthesia in patients undergoing major spine surgery	Wrong comparison – dexmedetomidine
Nitta et al. 2013	Combination of oral clonidine and intravenous low-dose ketamine reduces the consumption of postoperative patient-controlled analgesia morphine after spine surgery	Wrong comparison – active protocols
Khandelwal et al. 2024	A comparative study to assess the hypotensive property of dexmedetomidine and clonidine in patients undergoing lumbar spine surgery	Wrong intervention – IV clonidine
Reena & Vikram 2017	Comparative evaluation of Clonidine and magnesium sulfate infusions upon intraoperative hemodynamics and anesthetic consumption, and postoperative recovery profile in lumbar spine surgery: A prospective, randomized, placebo controlled, double-blind study	Wrong intervention – IV clonidine
Rajkumar et al. 2016	The efficacy of intravenous clonidine premedication to reduce surgical area bleeding in posterior lumbar spine fusion surgery	Wrong intervention – IV clonidine

Table S4. Detailed summary of anesthesia and surgical protocols of the included trials.

Study ID	Anesthesia protocol	Surgical protocol
Aezi et al. 2023	Patients received premedication with midazolam (0.01 mg/kg) and fentanyl (0.02 mg/kg). Induction was achieved using IV thiopental (4–6 mg/kg) and atracurium (0.5 mg/kg), followed by intubation with a 7.5–8 tube. Maintenance included IV morphine (0.1 mg/kg) and atracurium (0.01 mg/kg), combined with 50% nitrous oxide in oxygen and isoflurane (0.5–1.5 MAC).	Not reported
Aghazadeh & Mahdkhah 2018	Not reported	Not reported
Aiad et al. 2024	After standard monitoring and IV fluids, anesthesia was induced with propofol (2 mg/kg), fentanyl (2 µg/kg), lidocaine (1 mg/kg), and cisatracurium (0.15 mg/kg) for intubation. Maintenance was achieved with isoflurane (1–1.2%) in air/oxygen (1:1), ventilation adjusted to keep $ETCO_2$ 30–35 mmHg, with additional cisatracurium top-ups. Granisetron (1 mg IV) was given for PONV prophylaxis.	Not reported
Ali et al. 2018	After IV access and fluids (20 ml/kg), patients were pre-oxygenated (100% O_2 for 2–3 min), then received fentanyl (2 µg/kg). Induction was done with propofol (1.5–2.5 mg/kg) and cisatracurium (0.15–0.2 mg/kg), followed by intubation. Maintenance used isoflurane (1.5 MAC) with cisatracurium 0.02–0.03 mg/kg every 30 min, plus controlled ventilation (TV 8 ml/kg, RR 12/min, $ETCO_2$ 30–35 mmHg). Reversal at the end was achieved with neostigmine (0.05 mg/kg) and atropine (1 mg), followed by extubation and transfer to recovery once fully conscious.	Not reported
Anvari et al. 2012	After IV premedication with midazolam (0.05 mg/kg) and fentanyl (3 µg/kg), anesthesia was induced with propofol (2 mg/kg) and atracurium (0.5 mg/kg). Maintenance was with continuous propofol (100 µg/kg/min), remifentanil (0.1–1 µg/kg/min, titrated to MAP 60–70 mmHg), and atracurium (10 mg every 30 min); nitroglycerin was added if target hypotension was not achieved. Patients were ventilated with TV 10 mL/kg, RR 10/min, I:E 1.3, adjusted to normocarbia. Additional fentanyl (1 µg/kg) was given before closure, with Ringer's lactate and saline as intraoperative fluids.	Not reported
Bala et ak. 2019	After IV access and standard monitoring, anesthesia was induced with fentanyl (2 µg/kg), thiopentone (sleep dose), and vecuronium (0.1 mg/kg) for intubation. Maintenance was achieved with isoflurane (titrated to BIS 40–60) in O_2 (35%) and N_2O (65%), with intermittent vecuronium (0.02 mg/kg). The incision site was infiltrated locally with 1% lignocaine + adrenaline (1:200,000). Surgery was performed in the prone position (open posterior approach). Fluids and transfusion were given as needed. At closure, isoflurane and N_2O were stopped, and neuromuscular blockade was reversed with neostigmine (50 µg/kg) + glycopyrrolate (10 µg/kg), followed by extubation.	Surgery was performed via the open and posterior approach in the prone position. Laminectomy of required levels was performed to perform cord decompression followed by fixation of the spine using pedicle and screw.
Janatmakan et al. 2019	Standard monitoring (ECG, SpO_2, NIBP) and IV fluids (Ringer's 7 mL/kg) were started. Induction was done with midazolam (0.05 mg/kg), fentanyl (3 µg/kg), sodium thiopental	Not reported

	(5 mg/kg), and atracurium (0.5 mg/kg). Maintenance used 1% isoflurane with O_2/N_2O mixture, and a radial arterial line was placed for invasive BP monitoring. Hemodynamics (HR, SBP, DBP, MAP) were recorded at baseline, post-intubation, 15, 30, and 60 minutes after incision, and after extubation.	
Naghipour et al. 2024	All patients received midazolam (2 mg IV) + fentanyl (100 µg IV), followed by induction with propofol (2 mg/kg), cisatracurium (0.15 mg/kg), and lidocaine (1 mg/kg). After 3 minutes, patients were intubated with a cuffed spiral tube (8 mm men, 7.5 mm women). Maintenance consisted of isoflurane (1%) with N_2O/O_2 (3 L/min each) plus remifentanil infusion (0.05 µg/kg/min). Hemodynamics (BP, HR, O_2 sat, ECG) were recorded at baseline, post-induction, post-intubation, after incision, and every 15 minutes until recovery. Rescue medication was planned if BP >150/100 despite anesthesia.	Not reported

Figure S1. Leave-one-out sensitivity analysis of the **[A]** duration of surgery (minutes), and **[B]** estimated blood loss (mL).

[A]

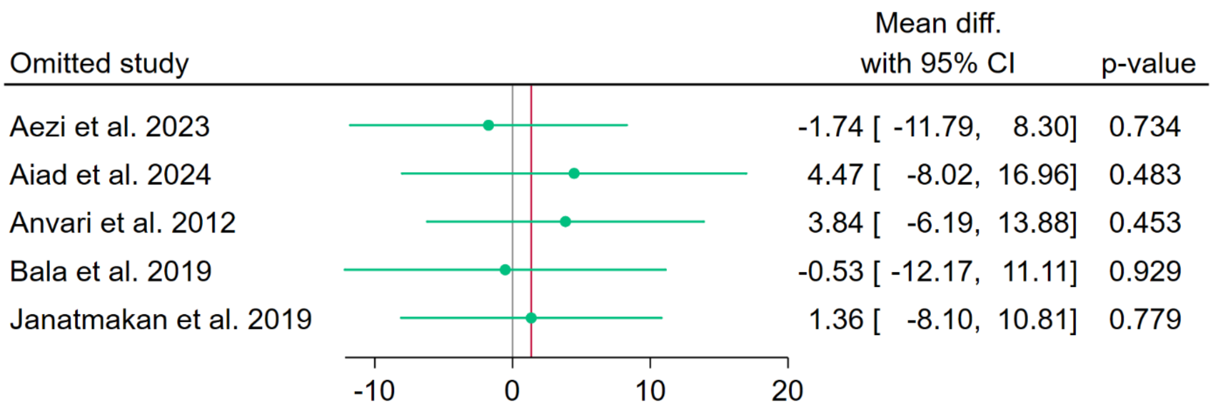

[B]

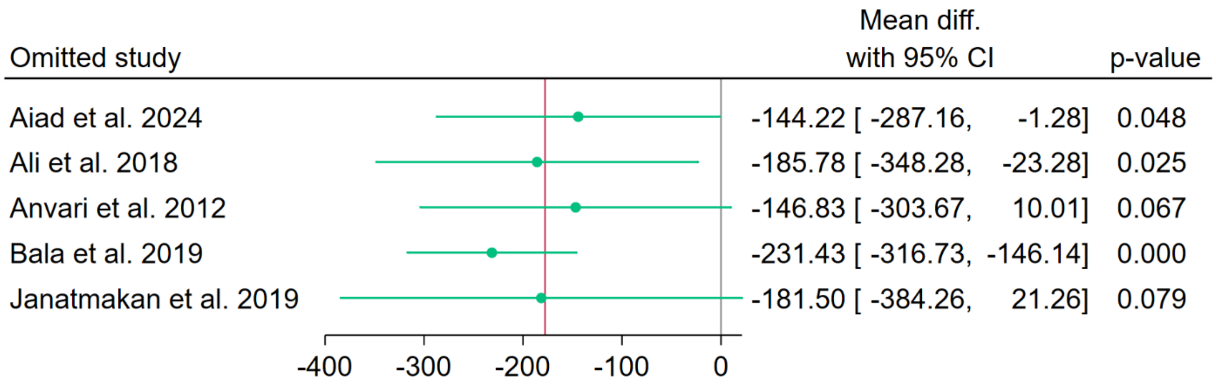

Figure S2. Leave-one-out sensitivity analysis of the intraoperative **[A]** heart rate (bpm), **[B]** mean arterial pressure (mmHg), and **[C]** total postoperative opioid consumption.

[A]

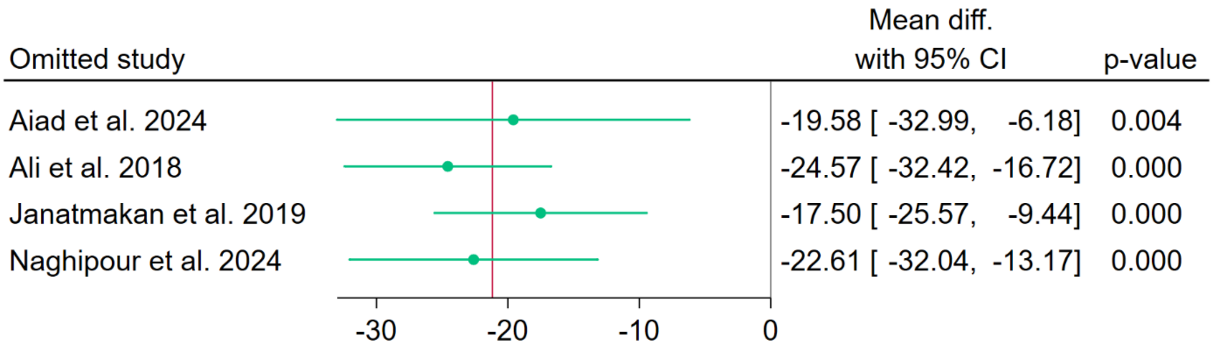

[B]

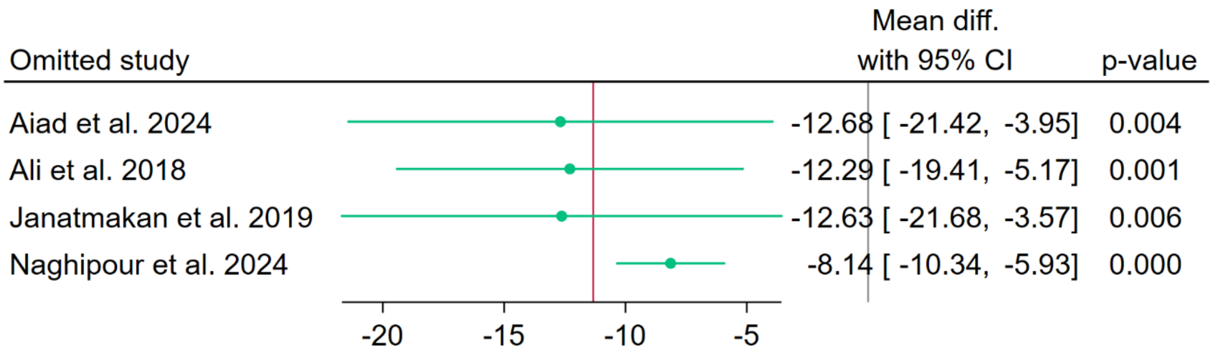

[C]

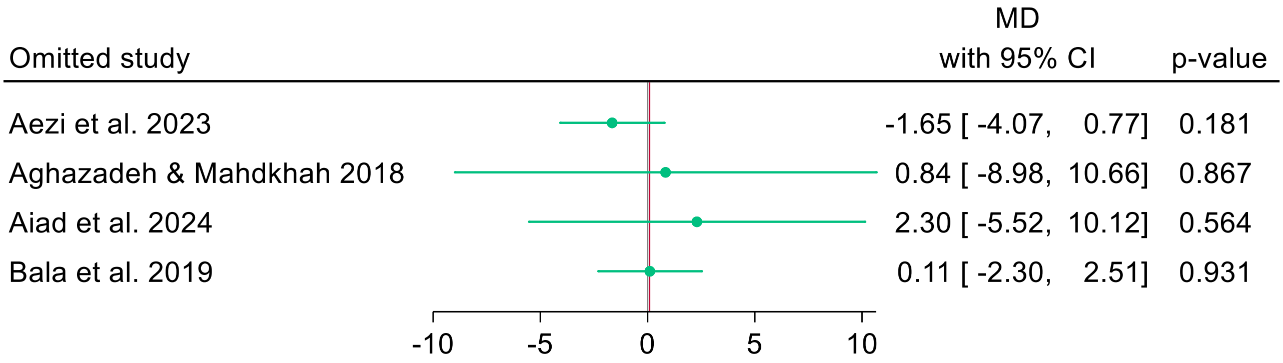

Figure S3. Meta-analysis of the surgeons' satisfaction score (3-points). CI = confidence interval.

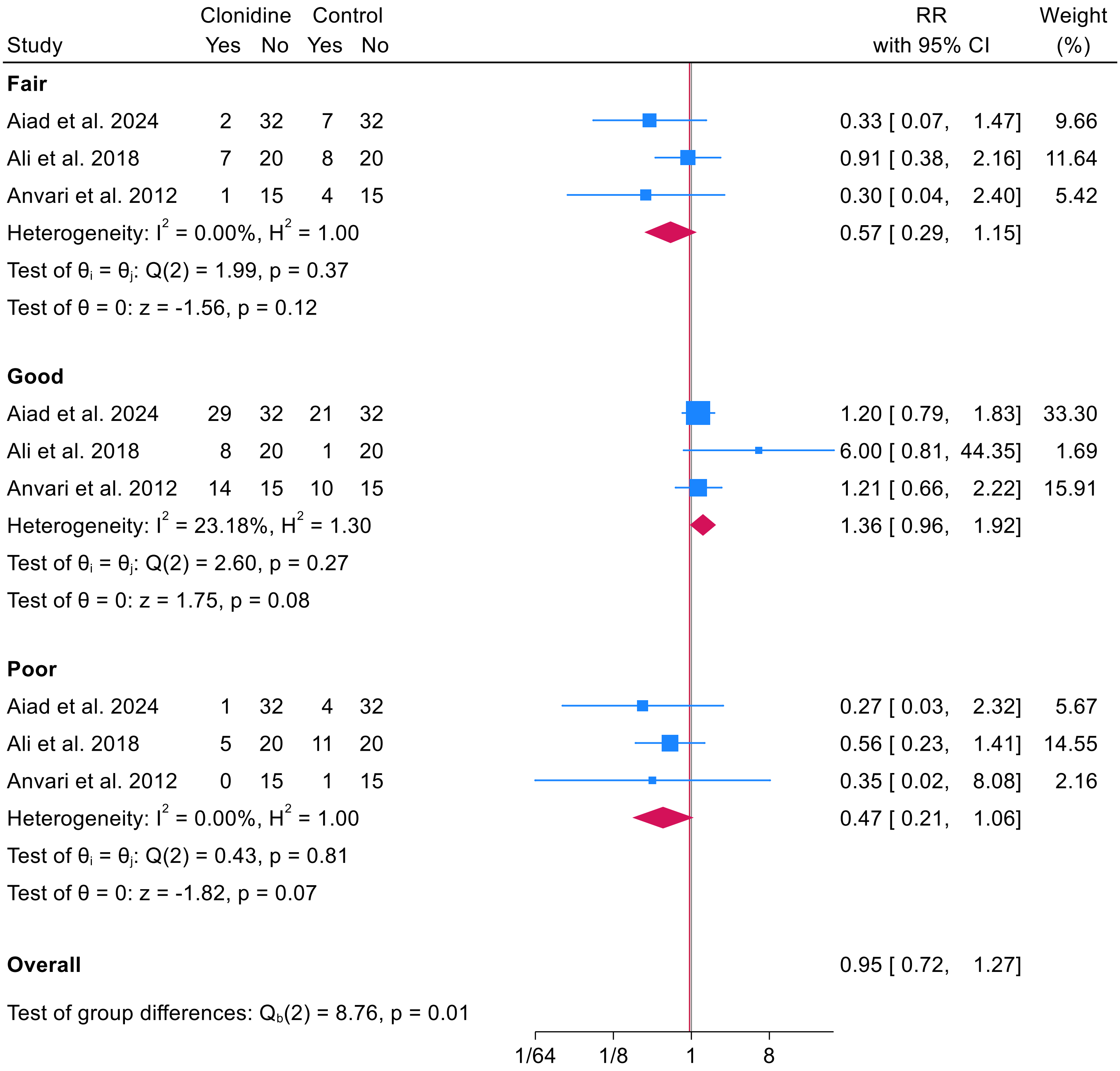

Supplement: Supplementary file 1 [file jcm-15-05270-s001.zip › jcm-4366530-supplementary.pdf]
